# Relationships between Interleukin 18 -607 C/A and -137 G/C, Osteopontin -9250 C/T Genetic Polymorphisms and Systemic Inflammatory Response Syndrome in Coronary Artery Bypass Graft Surgery

**DOI:** 10.3390/medicina60050724

**Published:** 2024-04-27

**Authors:** Serdal Kenan Köse, Bensu Karahilal, Başak Engin, Gülçin Aydoğdu, Seyhan Yağar, Kaan Orhan

**Affiliations:** 1 Department of Biostatistics, Faculty of Medicine, Ankara University, 06620 Ankara, Turkey; kose@medicine.ankara.edu.tr; 2 Department of Pharmaceutical Toxicology, Gazi University Faculty of Pharmacy, 06330 Ankara, Turkey; bensuka@gmail.com (B.K.); abengin@gmail.com (B.E.); 3 Department of Biostatistics, Faculty of Medicine, Hitit University, 19030 Çorum, Turkey; gulcinaydogdu06@gmail.com; 4 Department of Anesthesiology, Ankara City Hospital, 06800 Ankara, Turkey; 5 Department of Dentomaxillofacial Radiology, Faculty of Dentistry, Ankara University, 06620 Ankara, Turkey; call53@yahoo.com; 6 Department of Dental and Maxillofacial Radiodiagnostics, Medical University of Lublin, 20-059 Lublin, Poland; 7 Medical Design Application and Research Center (MEDITAM), Ankara University, 06620 Ankara, Turkey

**Keywords:** systemic inflammatory response syndrome, interleukin 18 -137 G/C genetic polymorphism, interleukin 18 -607 C/A genetic polymorphism, osteopontin 9250 C/T genetic polymorphism, CABG surgery

## Abstract

*Background and Objectives:* Systemic inflammatory response syndrome (SIRS) is one of the most significant complications after on-pump heart surgery procedures. High cytokine levels have been shown after open-heart surgeries and a genetic predisposition seems to be an important underlying modulatory characteristic for SIRS. To investigate the association between interleukin 18 -607 C/A, interleukin 18 -137 G/C and osteopontin 9250 C/T genetic polymorphisms and SIRS in on-pump CABG patients. *Materials and Methods:* Two hundred consecutive elective on-pump CABG patients were recruited prospectively to the study. Genomic DNA was extracted from whole blood and genotyping was determined by sequence specific PCR or PCR-RFLP methods for related polymorphisms. *Results:* SIRS incidence was 60.2%, 38.1%, 18.9% on postoperative day 1, 2 and 3, respectively, in the whole study population. The SIRS rate on the second postoperative day was 13% and 43.4%, respectively, in osteopontin 9250 C/T T allele non-carriers and carriers (*p* = 0.004). WBC (White Blood Cell) counts were higher on day 2 and 3 in osteopontin 9250 C/T T allele carriers compared to non-carriers (day 2; 12.7 ± 4 vs. 10.5 ± 2.4 (*p* = 0.015), day 3; 11.8 ± 4 vs. 9.1 ± 4.7 (*p* = 0.035)). The average ICU stay was 3.1 ± 7.4, 1.28 ± 0.97 for IL 18-137 G/C C allele carriers and non-carriers, respectively (*p* = 0.003), and in the IL 18-137 G/C C allele carriers, SIRS developed in 42.2% by the second postoperative day whereas the rate was 57.8% in non-carriers (*p* = 0.025). *Conclusions*: The current research revealed a possible link between osteopontin 9250 C/T and IL18-137 G/C genetic polymorphism and SIRS and morbidity in on-pump CABG patients.

## 1. Introduction

Systemic inflammatory response syndrome (SIRS), which has a multifactorial etiology, is one of the most significant complications after on-pump heart surgery procedures. The main causes of inflammation during open-heart surgery are surgical trauma, cardiopulmonary bypass (CPB) and reperfusion injury [1] Clinically, the spectrum of SIRS spans from the absence of symptoms to multiple organ failure and mortality, necessitating further investigation into the underlying determinants of this wide-ranging variability. High cytokine levels have been shown after open-heart surgeries and a genetic predisposition is an important modulatory characteristic of SIRS. The pivotal role of genetic coding regions in pro-inflammatory and anti-inflammatory mediators to individual variations in host responses during infection and inflammation is now an indisputable fact [2].

Interleukin (IL)-18, is a key cytokine regulator involved in adaptive and innate immune responses. Several studies have suggested a potential association between the IL-18 promoter -137 G/C polymorphism and alterations in IL-18 expression, thereby potentially influencing the onset of cardiovascular disease (CVD). Furthermore, abundant evidence in the literature underscores the involvement of IL-18 in diverse infectious, metabolic and inflammatory conditions [2,3,4]. 

Osteopontin (OPN), a matrix extracellular glyco-phosphoprotein, is found in various tissues such as epithelium-lined tissues, kidney, bones and teeth. OPN is involved in bone remodeling, cardiac remodeling, calcification, immune response, inflammation, regulation of cell adhesion, migration and cell survival. In non-bone tissues, OPN expression occurs only in response to stimuli such as inflammation. During inflammation, OPN is secreted by T-lymphocytes and activates macrophages. Furthermore, it plays significant roles in atherosclerosis development [5,6]. The exact role of osteopontin in vivo immune responses is partly unclear since it is believed to mainly act as a pro-inflammatory cytokine through chemo-attracting monocytes/macrophages [7]. Recent publications have highlighted the multifaceted nature of osteopontin (OPN), which exhibits a dichotomous phenotype that is contingent upon the pathological context. Acute elevations in OPN levels have been demonstrated to confer protective effects, including the facilitation of wound healing, the promotion of neovascularization and the mitigation of vascular calcification. Conversely, chronic upregulation of OPN has been linked to a heightened risk of adverse cardiovascular events, independent of conventional cardiovascular risk factors. Several studies have also prognosticated a poor clinical outcome in such circumstances [8].

Understanding the pathophysiology and underlying determinants of interindividual variability in systemic inflammatory response syndrome (SIRS) is imperative for the identification of high-risk patients and the development of novel preventative or therapeutic strategies. In this study, we explored the potential association between IL-18 -607 C/A, IL-18 -137 G/C and OPN -9250 C/T genetic polymorphisms and SIRS in patients undergoing on-pump coronary artery bypass grafting (CABG). IL-18 polymorphisms, known for their potential impact on post-open-heart surgery inflammation, were selected and reassessed based on a comprehensive literature review that considered their effects. The investigation into OPN genetic polymorphism aimed to address the gap in the literature, as informed by Vaschetto et al.’s study [9], which reported elevated OPN levels in SIRS patients. Notably, there is a dearth of similar research in the existing literature, underscoring the novelty and significance of our inquiry.

## 2. Materials and Methods

### 2.1. Study Design

After IRB approval (20.10.2016/435) and receiving written informed consent, consecutive 200 elective on-pump CABG patients were recruited prospectively to the study between November 2016 and November 2017. Intense inflammatory response shown after open heart surgeries was the main reason to select this population. For the purpose of eliminating possible influence from ethnic heterogeneity, only Caucasians were enrolled in this study population. Patients completed standardized health questionnaires related to their medical history, as well as to exposure and lifestyle factors including smoking habits, drug consumption and pre-existing medical conditions. This comprehensive data collection aimed to account for any potential confounding effects arising from these factors during the analysis.

The inclusion criterion for participation in the study was undergoing elective CABG surgery. Exclusion criteria were repeat procedures, preoperative inflammatory disorders, infections, malignancies, chronic treatment with steroids or immunosuppressive drugs, hepatic dysfunction (twice the normal level of liver enzymes), renal dysfunction (blood urea nitrogen > 30 mg/dL or creatinine > 2 mg/dL). 

Demographic and clinical characteristics, including the presence of diabetes mellitus, hyperlipidemia, hypertension, chronic obstructive pulmonary disease, central nervous system dysfunctions, tobacco addiction, preoperative medications, intraoperative variables and the postoperative course over a 3-day period, were recorded. Routine hematologic and biochemical laboratory results were recorded preoperatively and for 3 days postoperatively. 

In the operating room, patients underwent SpO_2_, invasive arterial and central venous pressure monitoring. All patients underwent standardized anesthesia (fentanyl, midazolam, sevoflurane and rocuronium bromide). CABG was carried out through median sternotomy, using a standardized CPB and cardioplegia technique. CPB was carried out with a 40 μm arterial blood filter, hollow fiber membrane oxygenator (Sorin Dideco Avant D 903, Sorin Group GmbH, Munich, Germany), heparin coated circuits and a non-pulsatile roller pump (Jostra HL20, Jostra Medizintechnik AG, Hirrlingen, Germany). Mean arterial pressure was maintained between 50–60 mmHg and blood flow was kept at 2.0–2.4 L/min per m^2^. Moderate systemic hypothermia (32–34 °C) was applied. Intra-operative risk factors such as cross clamping, CPB and operation duration and applied positive inotropics were recorded.

The diagnosis of SIRS was made according to the American College of Chest Physicians and the Society of Critical Care Medicine SIRS criteria [8]. SIRS is recognized upon the presence of two or more of the following criteria: body temperature > 38 °C or <36 °C, heartbeat > 90/min, tachypnea > 20/min or PaCO_2_ < 32 mmHg and abnormal white blood cell count (WBC > 12,000 cells/mm^3^ or <4000 cells/mm^3^ or >10% bands) after the surgery for 3 days. Respiratory rate or PaCO_2_ < 32 mmHg criteria were not used if the patient was mechanically ventilated.

### 2.2. DNA Extraction and Genotyping Analysis 

Five-milliliter venous blood samples were drawn into an EDTA coated vacuum tube at the beginning of the operation. Genomic DNA was extracted from whole blood using a sodium perchlorate/chloroform extraction method previously described by Kocabaş and Karahalil [10]. Genotyping was carried out using sequence specific polymerase chain reaction (PCR) or polymerase chain reaction and restriction fragment length polymorphism (PCR-RFLP) methods. 

### 2.3. IL-18 Gene Promoter Polymorphisms

The single nucleotide polymorphisms at positions -607 (C/A) and -137 (G/C) in the promoter region of the human IL-18 gene, which is located in chromosome 11q22.2-q22.3, were analyzed using the sequence specific PCR method using genomic DNA. For the position -607 specific PCR, a common reverse primer (-607 R: 5′-TAACCTCATTCAGGACTTC C-3′) and two sequence-specific forward primers (-607 FC: 5′-GTTGCAGAAAGTGTAAAAATTATTAC-3′ and -607 FA: 5′-GTTGCAGAAAGTGTAAAAATTATTAA-3′) were used to amplify the 196 bp product. A control forward primer (-607 CTRL: 5′-CTTTGCTATCATTCCAGGAA-3′) was used to amplify the 301 bp fragment covering the polymorphic site as an internal positive amplification control. The PCR was performed in 20 µL volume containing 0.4 µM of one sequence specific primer and -607 R, 0.13 µM of -607 CTRL, 10 mM Thermopol buffer, 1.0 U AmpliTaq Polymerase and 30 ng genomic DNA. For the position -137-specific PCR, a common reverse primer (137 R: 5′-AGGAGGGCAAAATGCACTGG-3′) and two sequence-specific forward primers (-137 FG: 5′-CCCCAACTTTTACGGAAGAAAAG-3′ and -137 FC: 5′-CCCCAACTTTTACGGAAGAAAAC-3′) were used to amplify the 261 bp product. A control forward primer (-137 CTRL: 5′-CCAATAGGACTGATTATTCCGCA-3′) was used to amplify the 446 bp fragment covering the polymorphic site as an internal positive amplification control. The PCR was performed in 20 µL volume containing 0.4 µM of one sequence specific primer and 137 R, 0.13 µM of -137 CTRL, 10 mM Thermopol buffer, 1.0 U AmpliTaq Polymerase and 30 ng genomic DNA [11].

### 2.4. OPN 9250 CT Gene Polymorphism

OPN 9250 (C/T) gene polymorphism localized in exon 7 of the OPN gene in the chromosome 4q13 [12] was detected by PCR-RFLP. A sense primer (5′-TACCCTGATGCTACAGACGAGG-3′) and antisense primer (5′-CTGACTATCAATCACATCGGAATG-3′) were used to amplify a 252-bp product as described by Kikuchi et al. [13]. The amplified DNA was digested with Alu I restriction enzyme and the cleaved fragments were analyzed by electrophoresis in a 2% gel with ethidium bromide [14].

### 2.5. Statistical Analysis

Statistical analyses were performed using the SPSS for Windows version 11.5 (Chicago, IL, USA). Data were expressed as mean ± SD for normally distributed continuous variables, frequencies and percentages for categorical variables. Chi-square or Fisher Exact tests are used to detect differences between non-carrier and carrier groups for categorical data. The difference between two groups for continuous variables was evaluated by Student’s *t* test. In all analyses *p* value of <0.05 is considered as statistically significant. 

## 3. Results

The clinical characteristics of the 200 participants and their genotype groups, which are classified as mutant allele carrier or non-carrier, are presented in Table 1. As a result of comparisons between carriers and non-carriers for each allele, no statistically significant differences were found in terms of patient characteristics, pre-existing conditions or medications (Table 1). The genotype distribution of this study population and a healthy Caucasian population are shown in Table 2. The present study population’s Osteopontin 9250 C/T (For AA genotype: study population 6%, healthy Caucasian 14.6%) and IL18 -607 C/A allele frequencies (For CT genotype: study population 68.5%, healthy Caucasian 22.2%) differed significantly from the healthy Caucasian population (*p* < 0.05) [15,16].

This investigation’s primary aim was to clarify the relationship between related genetic polymorphisms and SIRS occurrence; as such, using ICU stay as an outcome would be senseless with our sample size due to the mortality rate. Many authors, therefore, have used prolonged ICU stay as a measure of adverse outcome [2]. 

SIRS incidence was 60.2%, 38.1%, 18.9% on postoperative day 1, 2 and 3, respectively, in the whole study population. The SIRS rates on the second postoperative day were 13% and 43.7%, respectively, in the Osteopontin 9250 C/T T allele non-carriers and carriers (*p* = 0.004) (Table 3). The differences between the incidence of SIRS on the first, second and third days for those with a pre-existing condition were also examined. According to this, only the day 2 SIRS incidence in those without hypelipidemia was statistically significantly higher than in those with hyperlipidemia (HL(+): 62.5%, HL(−): 23.9%, *p* = 0.001). There were no statistically significant differences in SIRS incidences on the first, second and third days according to smoking habit, diabetes, hypertension, cOPD, neurologic disorders or gender. WBC counts (×10^9^/L) were higher in day 2 and 3 in Osteopontin 9250 C/T T allele carriers compared to the non-carriers (day 2; 12.7 ± 4 vs. 10.5 ± 2.4 *p* = 0.015, day 3; 11.8 ± 4 vs. 9.1 ± 4.7 *p* = 0.035). Cross-clamping time (65.8 ± 26.9 min, 63.2 ± 24.9 min, *p* = 0.487), CPB time (104.8 ± 41.4 min, 97.4 ± 33.9 min *p* = 0.227) and operation duration (266 ± 90.6 min, 259 ± 56.5 min *p* = 0.220) were similar for T allele non-carriers and carriers (Table 4).

The length of ICU stay was 3.1 ± 7.4, 1.28 ± 0.97 for IL 18-137 G/C C allele carriers and non-carriers, respectively (Table 4, *p* = 0.003); 48% of the IL 18-137 G/C C allele carriers developed SIRS by the second postoperative day, whereas the rate was 31.7% in the non-carriers (Table 3, *p* = 0.025). Cross-clamping time (66.5 ± 27.3 min, 61.5 ± 22.9 min *p* > 0.05), CPB time (101.8 ± 38.3 min, 96.2 ± 31.8 min *p* > 0.05) and operation duration (270.2 ± 67.1 min, 252 ± 57.4 min *p* > 0.05) were similar for C allele non-carriers and carriers.

ICU duration and SIRS ratio were similar according to the IL 18-607 CA genotype. 

The in-hospital reoperation rate was 6% (*n* = 12). Three of these patients developed sepsis, and among them two patients died.

In our study population, twenty-eight (14%) patients developed an infectious complication and nine (4.5%) patients developed septicemia during their hospital stay. Patients that had SIRS criteria in day 3 showed high infectious complications ratios (73.6%, *p* < 0.001). Infective patients’ genotype distributionds were eight A allele non-carriers and twenty carriers for IL -607 CA (*p* = 0.526); fifteen C allele non-carriers and thirteen carriers for IL -137 GC (*p* = 0.292); and four T allele non-carriers and twenty-four carriers for OPN 9250 CT (*p* = 0.577). There were no significant relationships between infection and sepsis incidence and any of the studied genotype regions. 

## 4. Discussion

To our knowledge, this is the first study that has investigated the relationship between OPN genetic polymorphism and SIRS. The current research a revealed possible link between OPN -9250 C/T and IL18-137 G/C genetic polymorphism and SIRS and morbidity in on-pump CABG patients. A secondary, but still considerable, finding of present study is that the OPN -9250 C/T genetic polymorphism seems to play a role in coronary artery atherosclerosis. As shown previously, OPN is an important marker for atherosclerotic vascular diseases [17,18]. Our study population’s higher level of OPN 9250 T allele (mutant allele) frequency compared to the healthy Caucasian population is suggestive of its negative effects on coronary atherosclerosis formation [18].

Modern hypotheses propose that injury leads to synchronous, opposite responses, pro-inflammatory responses (SIRS) and anti-inflammatory responses. The dominancy of pro-inflammatory side effects may precipitate organ dysfunction [19]. Thus, the balance between pro-inflammatory and anti-inflammatory cytokines seems like the key pathway for controlling clinical response. In our study, three different SNPs regions, which code for pro-inflammatory cytokines, were studied, therefore it is hard to analyze their clinical effects without any data on the converse pathway or whole genome; as such, our findings can be taken into to account as a signal in a rather complex immune system genome.

There are limited studies focusing on SIRS as defined by clinical criteria after open heart surgery, and its relevance to clinical outcomes and morbidity [20]. In the present study, SIRS occurrence was 60.2% on the first postoperative day in CABG patients. There have been two large studies that have defined SIRS with clinical criteria in different types of cardiac operations; in 2019, its incidence was 28.3% at 24 h [21], whereas the previous study showed a high incidence of 96.2% within 24 h of ICU admission [22]. To our knowledge there is no study reporting the daily course of SIRS incidence following cardiac surgery; MacCallum and colleagues [22] have followed up on the daily criteria that promote SIRS positivity, but they did not report the incidence. The present study shows the daily course of SIRS after CABG surgery, and the correlation of SIRS with genotype has been shown to only be present on the second postoperative day. There may be several reasons for this finding. First, different molecules may be responsible for triggering early inflammatory responses (first 24 h) and fail to differentiate their discrete effects. Second, there may be some delay between the onset of injury and the expression of specific candidate genes. Larger scale SIRS studies are needed to improve the understanding of how genetic variability influences the host responses on a daily basis.

Our study showed that patients who had SIRS on the third postoperative day were at a high risk of infection (73.6%). In addition, if SIRS still continues on the third day, this indicates a predisposition to infection. 

Osteopontin is up-regulated in response to injury, stress and inflammation in miscellaneous cells, and plays roles in homeostasis, angiogenesis, wound healing and immune reactions [23]. Osteopontin is a critical cytokine for neutrophil chemotaxis, response to infection and enhancing bacterial clearance and the migration of neutrophils, and other leucocytes may contribute to its pro-inflammatory action [24]. It has been proved that OPN-deficient mice have diminished neutrophil responses [25]. In this study, the observed elevation in WBC count and high SIRS ratio on the second postoperative day in T allele carrier patients (heterozygote and mutant) seems to indicate an association with high OPN serum levels. Future investigations combining this genetic polymorphism and the OPN levels to show their effect on WBC count in inflammatory processes are essential to reveal this relationship. In general, OPN seems to be protective against infections; however, there are a few studies shown an opposite effect of OPN [26]. However, it is not reliable to argue for the long-term clinical effects of our findings due to the low infection and sepsis ratio of our study group. Future studies that can investigate the structure of OPN-coding genes in septic patients would shed light on this area.

IL 18 is a pro-inflammatory innate cytokine that induces INF-γ production through anti-CD3-Th1 cells [27]. The IL 18 precursor is present in a wide variety of cells, such as monocytes, macrophages, epithelial and endothelial cells in normal conditions, and can be easily secreted during inflammatory stimulations and it has been shown that its pleiotropic action depends on its cytokine environment [28]. The transcription activity of the IL 18 gene promoter fragment demonstrated that -607C/A and -137G/C are in the promoter region. A change from C to A at position -607 disrupts the cAMP-responsive element binding protein binding site, whereas a change from G to C at position -137 altered the histone H4 gene-specific transcription factor-1 nuclear factor binding site [29]. Intriguingly, these two SNPs have diverse clinical effects on individual diseases. 

In an early study, it was stated that IL 18 is involved in the inflammatory response and initiation of MODS (multiple organ dysfunction syndrome) following a cardiopulmonary bypass [30]. In our study, we identified an association between IL 18 137 G/C SNP and SIRS ratio and ICU and hospital stay, but there were no relationships with IL 18 607 C/A SNP. Contrary to Yamada’s study, in which they found that IL18 607 CA gene polymorphism mutation increases IL 18 expression and inflammation, we found no effect of IL 18 137 GC gene polymorphism on the ICU patients. As they reported, the ethnicity of the study population may cause this distinction [31]. Nevertheless, a study on Caucasian cardiac surgery patients showed no correlation between IL 18 137 G/C and IL 18 607 C/A SNPs and greater inflammation after surgery when IL 18 9545 T/G SNP caused aggravation [28]. Our results are concordant with a study on diabetes that showed that IL 18 137 G/C SNP is associated with a susceptibility to type 1 diabetes and that IL 18 607 C/A SNP is protective [32].

The primary limitation of our study is the absence of serum level measurements for related cytokines. However, a previous study demonstrated that genetic polymorphisms may not only influence the expression of the encoded cytokine but also impact other cytokines [33]. Additionally, our study is limited by the randomized patient recruitment, which precluded gender stratification. Furthermore, details regarding the duration of diabetes among diabetic patients and the duration of smoking among smokers were not provided. Additionally, information regarding medications used for chronic diseases was not fully elucidated. Moreover, our study was conducted at a single center. Although, beyond the scope of our study, we also explored variables affecting inflammation under the assumption that they might confound SIRS incidence. Notably, no significant effects of the gender or pre-existing conditions variables were detected, as mentioned in the results section.

## 5. Conclusions

The current research suggests a potential association between osteopontin 9250 C/T and IL18-137 G/C genetic polymorphisms and the occurrence of SIRS and morbidity in patients undergoing on-pump CABG. Notably, our study population exhibited a higher frequency of the OPN 9250 T allele (mutant allele) compared to the healthy Caucasian population, implying that it has potential adverse effects on coronary atherosclerosis formation. However, as our study focused on investigating three different single nucleotide polymorphism (SNP) regions encoding pro-inflammatory cytokines, translating their clinical implications without comprehensive data on the converse pathways or the entire genome is challenging. Hence, our findings should be interpreted as a signal within the intricate landscape of the immune system genome. It is important to note that our study was conducted exclusively within the Caucasian population; therefore, different results may emerge in diverse populations.

The findings from our study suggest a potential association between osteopontin T allele carrier status (heterozygous and mutant) and elevated White Blood Cell (WBC) count and a higher rate of systemic inflammatory response syndrome (SIRS) on the second postoperative day. These observations imply a potential link with increased osteopontin (OPN) serum levels. Future investigations that integrate genetic polymorphisms and OPN levels to elucidate their impact on WBC count during inflammatory processes will be instrumental in further understanding this relationship. Moreover, it is important to note that discussing the long-term clinical implications based solely on these results may not be reliable, emphasizing the need for further research in this area.

Future studies will investigate exploring the structure of OPN-encoding genes specifically in septic patients, which will provide valuable insights into this area of research.

## Figures and Tables

**Table 1:** Summary of epidemiological and clinical characteristics of the patients.

Characteristics	All Patients	OPN-T Allele Non-Carriers	OPN-T Allele Carriers	*p* Value	IL 18-607-A Allele Non-Carriers	IL 18-607-A Allele Carriers	*p* Value	IL 18-137-C Allele Non-Carriers	IL 18-137-C Allele Carriers	*p* Value
No of patients	200	28	172	NA	60	140	NA	94	106	NA
Gender (F/M)	36/164	6/22	30/143	0.188	12/48	24/116	0.360	17/77	18/88	0.520
Age (Years)	59.6 ± 8.9	60 ± 7.2	59.7 ± 9.1	0.876	59 ± 8.1	60 ± 9.1	0.286	56.9 ± 9.1	59.6 ± 8.6	0.352
Heights (cm)	166.8 ± 7	164.8 ± 8.2	167.1 ± 7.2	0.150	167.1 ± 6.7	166.7 ± 7.2	0.416	166.2 ± 6.8	167.4 ± 7.2	0.385
Weights (kg)	76.6 ± 11.8	74.6 ± 12.4	77.1 ± 11.8	0.366	76 ± 11.6	77 ± 12	0.975	76.1 ± 12.1	77.3 ± 11.7	0.308
BMI (kg/m^2^)	27.5 ± 4	27.3 ± 4.2	27.6 ± 4	0.799	27.2 ± 4.2	27.6 ± 3.9	0.333	27.5 ± 4	27.5 ± 4	0.351
Pre-existing conditions, *n* (%)										
Smoking habits	66 (33)	13 (46.4)	53 (30.8)	0.055	24 (40)	42 (30)	0.100	36 (38.2)	30 (28.3)	0.183
Diabetes	87 (43.5)	13 (46.4)	74 (43)	0.579	23 (38.3)	65 (46.4)	0.199	40 (44.6)	47 (44.3)	0.401
Hypertension	128 (64)	18 (64.2)	110(63.9)	0.504	39 (65)	89 (63.5)	0.520	61 (64.8)	67 (64.1)	0.521
Hyperlipidemia	93 (46.5)	13 (46.4)	80 (46.5)	0.552	28 (46.6)	65 (46.4)	0.542	43 (46.2)	50 (47.1)	0.540
COPD	36 (18)	7 (25)	29 (16.8)	0.246	16(26.6)	20 (14.2)	0.087	22 (23.4)	14 (13.2)	0.121
Neurologic disorder	22 (11)	3 (10.7)	19 (11)	0.521	6 (10)	16 (11.4)	0.528	9 (9.5	13 (13.7)	0.318
Medications, *n* (%)										
ASA	89 (44.5)	11 (39.2)	74 (43)	0.435	28 (46.6)	59 (42.1)	0.407	40 (42.5)	49 (46.2)	0.433
Clopidogrel	24 (12)	4 (14.2)	18 (10.4)	0.483	8 (13.3)	13 (9.2)	0.279	12 (12.7)	12 (11.3)	0.310
Statins	81 (40.5)	13 (46.4)	67 (38.9)	0.247	23 (38.3)	57 (40.7)	0.433	39 (41.4)	42 (39.6)	0.462
Beta-blockers	85 (42.5)	10 (35.7)	73 (42.4)	0.325	25 (41.6)	58 (41.4)	0.565	34 (36.1)	51 (48.1)	0.108
ACE inhibitors	72 (36)	13 (46.4)	63 (36.6)	0.171	20 (33.3)	52 (38)	0.408	32 (34)	40 (37.7)	0.390
Ca channel blockers	49 (24.5)	4 (14.2)	43 (25)	0.144	18 (30)	30 (21.4)	0.173	25 (26.5)	24 (22.6)	0.281

Abbreviations: BMI; Body Mass Index, COPD; Chronic Obstructive Pulmonary Disease, ASA; Aspirin, OPN; Osteopontin, IL; Interleukin.

**Table 2:** Frequency of genotypes in the study population and healthy Caucasian population.

Gene Polymorphism	Study Population *n* (%)	Healthy Caucasian *n* (%)	*p* Value
IL 18 -137 G/C			
GG	94 (47)	251(50)	0.313
GC	98 (49)	220 (43.8)	
CC	8 (4)	31 (6.2)	
IL 18 -607 C/A			
CC	60 (30)	166 (33.2)	0.020
CA	128 (64)	261 (52.2)	
AA	12 (6)	73 (14.6)	
OPN -9250 C/T			
CC	28 (14)	138 (76.7)	0.001
CT	137 (68.5)	40 (22.2)	

**Table 3:** SIRS ratios according to genotype.

SIRS	All Patients (*n* = 200)	OPN-T Allele Non-Carriers (*n* = 28)	OPN-T Allele Carriers (*n* = 172)	*p* Value	IL 18-607-A Allele Non-Carriers (*n* = 60)	IL 18-607-A Allele Carriers (*n* = 140)	*p* Value	IL 18-137-C Allele Non-Carriers (*n* = 94)	IL 18-137-C Allele Carriers (*n* = 106)	*p* Value
Day 1 (%)	60.2	47.8	62	0.145	60	60	0.520	58.5	61.3	0.423
Day 2 (%)	38.1	13	43.7	0.004 *	45	37.1	0.226	57.8	42.2	0.025 *
Day 3 (%)	18.9	13	20.8	0.293	18.3	20.7	0.489	25.5	15	0.068

* Statistically significant difference compared to other genotype groups.

**Table 4:** Operational data and WBC counts of the groups according to genotype.

	All Patients	OPN-T Allele Non-Carriers	OPN-T Allele Carriers	*p* Value	IL 18-607-A Allele Non-Carriers	IL 18-607-A Allele Carriers	*p* Value	IL 18-137-C Allele Non-Carriers	IL 18-137-C Allele Carriers	*p* Value
EF (%)	51.6 ± 10.6	50.8 ± 13.3	51.5 ± 10.3	0.507	51 ± 12.4	51.4 ± 9.9	0.071	49.7 ± 12	52.8 ± 9.3	0.173
X-Clamping time (min)	63.4 ± 25	65.8 ± 26.9	63.2 ± 24.9	0.487	67.3 ± 29.3	62.3 ± 23.1	0.027 *	66.5 ± 27.3	61.5 ± 22.9	0.134
CPB time (min)	98.3 ± 35	104.8 ± 41.4	97.4 ± 33.9	0.227	101.5 ± 41.5	97.7 ± 31.9	0.012 *	101.8 ± 38.3	96.2 ± 31.8	0.127
Operation time (min)	261.7 ± 61.9	266 ± 90.6	259 ± 56.5	0.220	271.4 ± 63.6	256.7 ± 61.8	0.213	270.2 ± 67.1	252.9 ± 57.4	0.464
Intubation time (h)	13.3 ± 14.1	10.7 ± 4.8	13.3 ± 14.7	0.322	11.8 ± 8.2	13.5 ± 15.4	0.267	12.3 ± 7.6	13.5 ± 17.3	0.228
ICU stay (day)	2.1 ± 5.1	1.7 ± 2.9	2.1 ± 5.4	0.556	2.6 ± 7.1	1.9 ± 4.1	0.106	3.1 ± 7.4	1.2 ± 0.9	0.001 *
Hospital stay (day)	7.8 ± 6.4	7.6 ± 4.1	7.7 ± 6.6	0.669	8.4 ± 8.5	7.6 ± 5.4	0.248	8.7 ± 8.3	7 ± 4.1	0.003 *
Preoperative WBC (×10^9^/L)	8.1 ± 1.9	7.4 ± 1.7	8.1 ± 1.9	0.091	7.8 ± 1.9	8.1 ± 1.9	0.315	8.0 ± 1.8	8.0 ± 2	0.865
Postoperative 1st day WBC (×10^9^/L)	11.5 ± 3.4	10.4 ± 2.9	11.8 ± 3.4	0.077	12.1 ± 3.9	11.3 ± 3.2	0.132	11.6 ± 3.5	11.4 ± 3.4	0.755
Postoperative 2nd day WBC (×10^9^/L)	12.3 ± 3.8	10.5 ± 2.4	12.7 ± 4	0.015 *	12.3 ± 3.9	12.3 ± 3.8	0.961	12.4 ± 3.3	12.3 ± 4.3	0.937
Postoperative 3rd day WBC (×10^9^/L)	11.3 ± 4.1	9.1 ± 4.7	11.8 ± 4	0.035 *	10.8 ± 3.3	11.6 ± 4.6	0.399	10.9 ± 3.8	11.8 ± 4.5	0.327

WBC: White Blood Cell; EF: ejection fraction; CPB: cardiopulmonary bypass; ICU: intensive care unit. * Statistically significant difference compared to other genotype groups.

## Data Availability

Data can be obtained by contacting the corresponding author.
